# Frontiers in effective control of problem parasites in beekeeping

**DOI:** 10.1016/j.ijppaw.2022.03.003

**Published:** 2022-03-10

**Authors:** Lewis J. Bartlett

**Affiliations:** Center for the Ecology of Infectious Disease, University of Georgia, Athens, GA, 30602, USA

**Keywords:** *Apis mellifera*, Parasite, IPM, Biological control, Regulation

## Abstract

Demand for better control of certain parasites in managed western honey bees (*Apis mellifera* L.) remains apparent amongst beekeepers in both Europe and North America, and is of widespread public, scientific, and agricultural concern. Academically, interest from numerous fields including veterinary sciences has led to many exemplary reviews of the parasites of honey bees and the treatment options available. However, summaries of current research frontiers in treating both novel and long-known parasites of managed honey bees are lacking. This review complements the currently comprehensive body of literature summarizing the effectiveness of parasite control in managed honey bees by outlining where significant gaps in development, implementation, and uptake lie, including integration into IPM frameworks and separation of cultural, biological, and chemical controls. In particular, I distinguish where challenges in identifying appropriate controls exist in the lab compared to where we encounter hurdles in technology transfer due to regulatory, economic, or cultural contexts. I overview how exciting frontiers in honey bee parasite control research are clearly demonstrated by the abundance of recent publications on novel control approaches, but also caution that temperance must be levied on the applied end of the research engine in believing that what can be achieved in a laboratory research environment can be quickly and effectively marketed for deployment in the field.

## Highlights

• Comprehensive, contemporary overviews of parasite control in beekeeping exist.
• Research in parasite control beekeeping is a lively, thriving field.
• Challenges can be separated into lab/research and regulatory/market.
• IPM concepts are apparent in beekeeping, but are arguably still in their infancy.

## Graphical abstract

**Figure:**
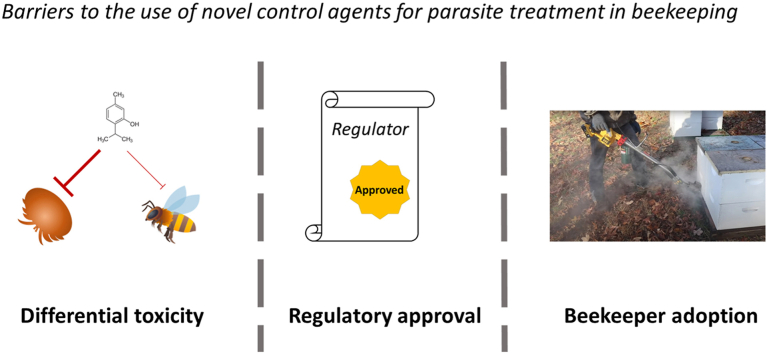

## 1 Rationale

### 1.1 Ecological, social, and economic context

Beekeeping is both economically and culturally valued, representing a traditional form of agriculture (Bingham 2006; Watson et al., 2011; Mace et al., 2012), and contributing substantially to both agricultural output via crop pollination (Delaplane, 2021; Klein et al., 2007; Knapp et al., 2017; Potts et al., 2016) as well as ecosystem resilience both within and outside its native range (Hung et al., 2018; Requier et al., 2019). Threats to beekeeping are therefore of agricultural, public, and (in certain circumstances) conservation concern. Efforts to understand the health of managed bee populations are reflected more widely amongst wild bees, which are similarly imperiled (Goulson et al., 2015; Potts et al., 2010).

A widely-recognised contributor to declines of bees both managed and wild is the (re-)emergence of parasites,^1^ especially those resulting from interspecific parasite transfer (Brosi et al., 2017; Manley et al., 2015; McMahon et al., 2018; Wilfert et al., 2016). Parasites interact synergistically with nutritional stress (Dolezal et al., 2019; Dolezal and Toth, 2018) and pesticide exposure (Annoscia et al., 2020; Dolezal et al., 2016; Sánchez-Bayo et al., 2016), placing them within a complex system of multiplicative contributors to observed declines in bee populations (Goulson et al., 2015). Notably, there is very substantial evidence demonstrating the spillover of honey bee parasites into wild bees (e.g. Graystock et al., 2016, 2013; Manley et al., 2019, 2015), further exacerbating the stressors of wild bees and explicitly linking the health of honey bees to wild bees beyond just shared environmental impacts.

Problems relating to parasite stressors in beekeeping are best described and most prevalent in parts of Europe and North America, where managed colony losses and poor honey bee health remain perceived as a pressing industry problem (El Agrebi et al., 2021; López-Uribe and Simone-Finstrom, 2019). Reflecting this, this manuscript focusses on parasite control in beekeeping in the context of North America and Europe, as these are regions with demonstrated sustained problems in beekeeping, which are arguably less prevalent in other regions. Table 1 summarises major pests based on Pasho et al. (2021), and which are used as illustrative emphasis in this review.

**Table 1:** Emphasis and relevant sections of this review corresponding to of major parasites in beekeeping based on Pasho et al., (2021).

Parasite (common name)	Parasite (binomial name)	Biological classification	Degree of concern	Relevant sections of this review
**American foulbrood/‘AFB'**	*Paenibacillus larvae*	Bacteria	Medium	Section 3.2
**European foulbrood/‘EFB'**	*Melissococcus plutonius*	Bacteria	Medium	Box 1; Section 3.2
**Varroa mite**	*Varroa destructor*	Ectoparasitic mite	High	Box 1; Box 2; Sections 2.4, 3.1, 4.1 & 4.2.
**Small hive beetle/‘SHB'**	*Aethina tumida*	Coleoptera	Varies/Regional	Sections 2.1, 2.3, 3.3
**Wax moths**	*Galleria mellonella*	Lepidoptera	Low	*Not discussed*
**Trachael mites**	*Acarapis woodi*	Endoparasitic mite	Low	*Not discussed*
**Chalkbrood**	*Ascophaera apis*	Fungi	Low	Section 3.2
**Stonebrood**	*Aspergillus* spp.	Fungi	Low	Section 3.2
**Nosema**	*Vairimorpha* spp.	Microsporidia	Medium	Section 2.0
**Deformed wing virus/‘DWV'**	*-*	Virus	High	Sections 2.2 & 2.3 (non-specific)
**Sacbrood virus**	*-*	Virus	Medium	Sections 2.2 & 2.3 (non-specific)
**Paralysis viruses (numerous)**	*-*	Virus	Medium	Sections 2.2 & 2.3 (non-specific)
**Black queen cell virus**	*-*	Virus	Low	Sections 2.2 & 2.3 (non-specific)

### 1.2 The need for treatments

The parasite burden placed on managed bees by pests and pathogens impacts the beekeeping industry directly, but is also a wider problem for agriculture in parts of the world where honeybees provide pollination services (Delaplane, 2021). This manifests in multiple ways; beekeepers suffering high overwintering losses struggle to grow colony counts to meet demand (Goodrich et al., 2019), and colonies weakened by ill-health (including parasites) are less effective supplementary pollinators (Dedej and Delaplane, 2003; Goodrich and Goodhue, 2020). Currently in the US demand from growers for pollination services for certain crops (e.g. almonds) outstrips convenient supply and poses an expensive problem which remains unaddressed due to colony loss. Inadequate parasite control commonly cited as a leading challenge in growing colony number (Aizen and Harder, 2009; Goodrich et al., 2019; Steinhauer et al., 2021); in Europe, estimates indicate that half of all growers similarly consider their yield to be pollinator limited (Breeze et al., 2019). Notably, there is evidence that colonies most closely associated with providing necessary in-demand monocrop pollination services are those that struggle more severely with inadequately controlled parasites (Bartlett et al., 2021; Welch et al., 2009). Improving the effectiveness of parasite control in beekeeping therefore has dual-measure benefit for the industry and wider agriculture: more colonies available, and better colonies on average. There is emerging evidence further that healthier bee colonies lead to lower spillover risk to adjacent wild bees (Burnham et al., 2021), although this is still an open line of research in the field.

Mounting contemporary evidence supports the assertion that treating bees for their parasites makes for better colony health (Haber et al., 2019; Hansen, 2021; Hernandez et al., 2022; Kulhanek et al., 2021; Steinhauer et al., 2021), this is a simple but important demonstration of an intuitive principle. We can therefore identify multiple frontiers in the effective treatment of parasites in beekeeping: improving uptake of current control strategies amongst beekeepers by identifying barriers to adoption of control strategies (see section 4.0) and developing new control strategies where current options are inadequate (see sections 2.0. and 3.0.). In doing so we improve beekeeping as an industry in and of itself, improve pollination in wider agriculture, and possibly improve wild bee health via reduced spillover.

### 1.3 Working treatments

Current treatment approaches to the litany of parasites which infect honey bees will differ between regulatory environments, with the USA typically being the slowest to approve novel treatments. Of note is the recent emphasis on requiring veterinarian involvement in honey bee health, related to their classification as livestock in parts of the world and tighter regulations on antibiotic use. Correspondingly, there are abundant recent efforts to summarize effective treatments for a variety of honey bee parasite infestations or infections including Kane and Faux (2021) and Applegate and Kyle (2021a). These recent reviews are specifically for the rapid education of veterinarians, which poses benefit to both veterinary training (Mayer, 2021) and interdisciplinary expansion of expertise in beekeeping pest and infectious disease management (Applegate and Kyle, 2021b). I point readers interested in simple primers on honey bee parasite diseases and an assessment of current treatment possibilities to Pasho et al. (2021) and Farone (2021), from Applegate and Kyle (2021a).

## 2 Current failures in treatments

Some honeybee parasites are yet to show sufficient susceptibility to any adequate, approved chemical controls, relying exclusively on cultural or biological treatments. Three notable but quite different examples include microsporidian infections by *Vairimorpha* (formerly *Nosema* – see Tokarev et al. (2020)), colony-level parasitism by the small hive beetle (SHB), *Aethina tumida*, and the large host of bee viruses known to correlate with colony loss or reduced productivity. In the case of *Vairimorpha*/*Nosema*, the only prescribed control agent used historically was fumagillin in the USA (Bailey, 1953; Burnham, 2019; Katznelson and Jamieson, 1952); significant work has however highlighted the ineffectiveness of this control against specific species of the microsporidia, leading to more severe outcomes of infection due displacement of the native *V. apis* by the invasive *V. ceranae* (Huang et al., 2013). Further problems in treatment arise when parasites interact with one another or contextual failures of control methods conspire to impair control; this creates a research frontier that often requires a challenging degree of appreciation for nuance, which may be particularly burdensome for extension or science-communication activities targeting practitioners.

### 2.1 Small hive beetles

In the case of SHB, parasite pressure has a strong environmental signature. SHB lifecycles include pupation underground in the soil, and their population growth is putatively limited by the conduciveness of the environment to this pupation step (Ellis et al., 2004; Meikle and Diaz, 2012). Warm, wet areas with specific soil types seem to favour SHB development, such as the southeast & gulf of the United States. Correspondingly, colony placement (such as on nonpermeable ground, e.g. concrete) or ensuring colonies are in full sunlight with little vegetation (to dry soils) helps prevent severe infestation. Other cultural controls typically relate to ‘good management’; in particular, ensuring bee populations are not spread ‘too thin’ in that hive bees are numerous enough to adequately guard and police the whole interior of the hive to prevent SHB infestation. Mechanical traps are also used to reasonable effect whereby traps with entrances large enough for SHBs to enter (or be actively chased into) but small enough that honey bees cannot enter are filled with a lethal agent (for example, vegetable oil or soap solutions). These cultural and mechanical controls are often adequate to prevent severe SHB outbreaks (Cuthbertson et al., 2013), but may be insufficient if colonies are weakened by other factors, where more aggressive treatments may then be necessary to prevent SHBs overwhelming weakened colonies. One biological control is commonly cited: the employment of entomopathogenic nematodes (EPNs) (Cabanillas and Elzen, 2006; Sanchez et al., 2021) to attack and kill SHB pupa in the soil. EPNs are a frontier in wider parasite agricultural control (Koppenhöfer et al., 2020), and have some success amongst beekeepers but are limited in effectiveness if colonies are frequently moved, if there are large numbers of adult SHBs already in the landscape, if suppliers cannot ensure live delivery (beekeepers may require a microscope of sufficient quality to check the EPNs are alive when purchased), or require significant infrastructural investment if beekeepers are raising their own EPNs. When infestations are apparent, chemical control options are rarely employed; a critical hurdle being the difficulty in identifying insecticides which are effective against SHBs (a coleopteran) but safe for honey bees (a hymenopteran). An obvious open niche in parasite control in beekeeping is bee-safe insecticides to combat other insect parasites such as SHBs, as many currently listed options are precluded for live colonies and instead intended for stored frames (Farone, 2021) or to be used external to colonies as a soil-drench.

### 2.2 Viruses

Treatment for viral infections remains challenging across all of human and veterinary medicine and agriculture; it is therefore perhaps unsurprising that management of viral infections or outbreaks in honey bees colonies also remains challenging. Much of ‘viral’ management in beekeeping focusses on the control of varroa mites, which are known to vector at least one honey bee virus (McMahon et al., 2018). Otherwise, treatment of suspected viral infection problems has few solutions. One well demonstrated factor is in ensuring adequate nutrition, and possibly access to plant secondary metabolites (Palmer-Young et al., 2017), via varied and abundant plant pollen, as reviewed by Dolezal and Toth (2018). ‘Treatment’ for viruses therefore currently encompasses a ‘honeybee holistic health’ approach, relying on low pesticide exposure and good nutrition, rather than acute or curative treatments. Colonies with chronic viral problems are likely to be requeened by a beekeeper, which may be understood as something of a panacea or ‘fix-all’ in beekeeping.

### 2.3 Interactions between parasites

Interactions between parasites can further inhibit the effectiveness of control methods; this can be illustrated by the combination of the two examples discussed above (SHBs and viruses). Manages honey bees in the U.S. are often supplementarily fed by beekeepers (Caron and Connor, 2013; Gemeda et al., 2018; Goodwin, 1986; Hoover et al., 2022; Mortensen et al., 2019; Sammataro and Weiss, 2013) and whilst the feeding of sugar solution is a common practice to aid in colony provision almost universally, pollen supplementation is rare in some regions such as the southeastern U.S. in part due to risks of severe SHB infestation. This is despite widespread evidence that polyfloral pollen is a critical component of bee health (DeGrandi-Hoffman et al., 2016; Hoover et al., 2022), for instance in reducing infectious pathogen burdens (Alaux et al., 2010; Bagheri and Mirzaie, 2019; LoCascio et al., 2019) including of viruses as discussed above (Dolezal et al., 2019). Inadequate control of parasite therefore (in this case SHB) can prevent the control of another (in this case, viruses).

### 2.4 Context-dependent failures in *varroa*

Many methods of control are context-dependent in their effectiveness. This is particularly the case for those relating to varroa. Numerous reviews either could be or already are dedicated purely to the effectiveness of different methods of control of varroa in honey bee colonies (see Box 1). Varroa have no shortage of possible chemical controls, but still remain a problem. One contributor to this is the evolution of resistance to highly selective acaricides by varroa populations. The biomolecular bases for resistance or susceptibility to numerous approved acaricides have recently been described; namely, resistance to the pyrethroid tau-fluvalinate is associated with specific site point mutations on the gene encoding the varroa voltage-gated sodium channel (Millán-Leiva et al., 2021), coumaphos resistance is linked to loss-of-function mutations in varroa cytochrome-P450 genes (Vlogiannitis et al., 2021), and the target molecule of amitraz leading to its differential toxicity to varroa and honey bees appears to be the octopamine receptor (Guo et al., 2021). Understanding resistance evolution in varroa is thus a frontier making rapid gains in the field; one likely future frontier is understand cross-resistance, or antagonistic susceptibility, of varroa to different acaricides based on mutations such as those recently characterized, as well as the broader costs of resistance in this system.

Box 1 **Integrative Pest Management in Beekeeping**

Integrative pest management (IPM) in beekeeping remains culturally popular amongst many hobbyist or sideline beekeepers, although its influence varies between practitioners. The core principles of IPM rely on a hierarchical approach to pest management where cultural and mechanical controls take precedence as preventative measures, followed by biological controls, reserving chemical treatments (organic or synthetic) as a last resort responsive control tactic; use of these acute approaches is based on monitoring of parasite prevalence, treating curatively only once parasite pressure surpasses some economic threshold. Preventative, prophylactic chemical control application is avoided, and control chemicals are in addition rotated to help reduce the rate at which parasites evolve resistance to control chemicals. In beekeeping, I highlight here two illustrative examples of where IPM intersects with bee parasite control: European foulbrood management and varroa control.

In the case of European foulbrood, historical recommendations in some parts of the world spanning back to the 1980's advised that beekeepers prophylactically treat with antibiotics (for example, oxytetracycline) as part of standard management to help control all foulbrood pathogens. Gradual change in policy from removing this recommendation towards actively discouraging antibiotic use, for example by requiring veterinarian approval (Farone, 2021), represents not only a wider shift in understanding of microbiome health and antibiotic resistance but also a shift towards an IPM-based approach to EFB among beekeepers. For some beekeepers practicing IPM, low levels of EFB may be seen as tolerable and monitored by eye, with appearances of EFB-killed larvae are understood as an indicator of other colony stressors. Correspondingly, cultural or biological treatments such as supplementary feeding, brood supplementation, or requeening are commonly used as first-line treatments prior to resorting to the application of antibiotics. However, in parallel to the case described below in varroa control, a challenge still remains in delivering effective and scalable screening or quantification protocols for the diversity of EFB strains that may present in colonies; this is discussed as part of recent efforts to develop a lateral flow monitoring protocol by Milbrath et al. (2021).

Varroa control is the current dominant concern in honey bee parasite control, including whether IPM approaches are suitable in beekeeping; IPM approaches for varroa control were recently reviewed in detail by Jack and Ellis (2021). Varroa are present in nearly all colonies worldwide (Wilfert et al., 2016), and become a problem when per-capita parasitism reaches certain thresholds. For beekeepers who do not treat according a preventative, prophylactic schedule (i.e. those who pursue an IPM approach) varroa must be monitored. This presents a challenge in its own right – ‘sticky screens’ can be placed on the bottom of colonies to monitor varroa fall, but these are low-accuracy measures that can't easily adjust for colony size. Per-capita mites can be measured by ‘washing’ phoretic mites from a cohort of adult honey bees; if done thoroughly (with ethanol solutions or soap water) this requires the sacrifice of a sample of adult bees (a cultural barrier) as well as counting of the number of adults (an economic barrier). An alternative used by some beekeepers is known as ‘sugar rolling’ where fine-ground powdered (icing) sugar is used to dislodge phoretic mites, allowing bees to be returned to the colony but sacrificing accuracy in both mites dislodged and known number of bees sampled (Gregorc et al., 2017). Perhaps a more contentious issue with ‘varroa IPM’ is balancing preventive vs curative chemical treatment, where chronically applied routine treatments may be capable of preventing varroa population growth (working essentially as a prophylactic application), but not capable of reducing parasite levels once a ‘problem threshold’ has been reached. In these cases, as may be seen for oxalic acid (Berry et al., 2022), beekeepers may see evidence of a treatment regime working that cannot be traditionally incorporated into an IPM-like framework. Berry et al. (2022) demonstrated how regular and repeated treatment with oxalic acid vapour while significant capped brood area is present in the colony may work as a prophylactic approach, preventing varroa population growth, however is not capable of reducing varroa populations once they pass some treatment threshold as would be used in a IPM framework. Of note however is the significant IPM-conforming investment and emphasis on breeding bees which are mite-resistant; this is a topic which has been recently reviewed in detail by numerous authors (Guichard et al., 2020; Le Conte et al., 2020; Mondet et al., 2020; Noël et al., 2020; Spivak and Danka, 2020; van Alphen and Fernhout, 2020).

Alt-text: Box 1

Box 2 **Tracking Failures**

Negative results are underreported broadly in science; in applied sciences, this presents as a problem when ineffective treatments are not sufficiently and widely reported as inadequate. Repeating high-investment work is a gross inefficiency, and tracking ineffective treatments requires better compilation of the literature. Parasite control in beekeeping is not lacking in productive research lines which, unfortunately, did not yield promising actionable recommendations. These failures in developing effective parasite controls span across IPM framework techniques (mechanical, biological, chemical); here, I highlight examples from the challenging arena of varroa control, where initially promising control methods have ultimately proven insufficient. It remains an important part of extension and scientist/practitioner communication to explain when approaches have been found to be ineffective.

Mechanical control: An early concept in reducing varroa reproductive potential was the idea that a ‘small cell’ restricts the physical space available to varroa to move and feed on developing bees, and that providing bees with frames imprinted with small cell patterning could impede varroa population growth. This intuitively appealing idea ultimately proved ineffective (Berry et al., 2010; Ellis et al., 2009; Seeley and Griffin, 2011).

Biological control: A old adage cites in biocontrol is that ‘the enemy of my enemy is my friend’ - this has been pursued in varroa control with initially promising prospects of using predatory pseudoscorpions to actively hunt varroa mites (van Toor et al., 2015). Despite being an attractive idea, this ultimately proved ineffective, as detailed by Rangel and Ward (2018).

Chemical controls: Across chemical prospecting papers exploring bee-safe acaricides is the abundant reporting of chemicals that are too dangerous to bees for deployment in the field or are simply ineffective acaricides. For example numerous potential acaricides screened by Bahreini et al. (2020) showed higher toxicity in bees than varroa mites, including bifenthrin, cyflumetofen, and fenpyroximate, to name a small few. While possibly frustrating for beekeepers, there is a side-benefit to identifying which prospective chemistries from elsewhere in agriculture may be unsafe for bees, as this may help prevent their application in pollinator-depending crops. This side-benefit reinforces the importance of reporting and collating ‘negative’ results for wider bee health in agriculture.

Alt-text: Box 2

Other (typically non-synthetic) options for varroa control are often limited due to the environment they are deployed in (Farone, 2021). For example, thymol (and similar essential oil treatments) and formic acid are effective at treating varroa infestations, but if given in too high doses at too high temperatures can fumigate the hive at an airborne concentration intolerable to the bees leading to either absconsion (common for thymol) or widespread brood death, compromised queen health, or colony loss (formic acid). Thus, effective treatment with these products may be unachievable in many warm climates during periods where varroa control is required. Oxalic acid poses less threat to the bees, but is incapable of penetrating wax-capped brood; this means that the majority of the varroa in a colony are not adequately exposed to an acute oxalic acid treatment if brood is present. In regions where there are extended periods of broodlessness oxalic acid can be easily and effectively applied (Al Toufailia et al., 2015; Jack et al., 2021, Jack et al., 2021). However in warmer regions this may not be possible without a forced brood break; this heavily limits its application. Additionally, there is an apparent effect of temperature and humidity in reducing acaricidal effectiveness of oxalic acid (Patricia et al., 2013). The core problem observed here is that the environmental conditions rendering many of these treatments impotent are shared: warm, tropical or subtropical weather where varroa already have longer population growth seasons (Smoliński et al., 2021) inhibits the effectiveness of multiple possible treatment options, either directly by shielding varroa due to honey bee phenology, or by increasing honey bee sensitivity to the control agent. Correspondingly, long-release or repeated oxalic applications have been developed and tested to circumvent these problems, with some promise (Maggi et al., 2016). However, these repeated applications (Berry et al., 2022) or deployment of proprietary products also carry their own economic barriers (cost of purchasing or labour), as discussed in section 4.1.

## 3 New developments in novel treatments

Efforts continue to address some of the unmet challenges in parasite control in beekeeping, with efforts to better combine current treatment approaches and improve efficiencies of already approved control tactics, particularly by combining multiple forms of control (see Box 1). In addition, new chemical application are being actively sought, and the ongoing frontier of biological control options maintains some promise, as described below.

### 3.1 Better methods of applying current treatments

In response to the inadequacy of particular non-synthetic controls in specific environments and the evolution of resistance to synthetic controls in parasite populations, namely varroa in both instances, significant effort both by practitioners and by researchers has been invested in improving or combining treatment options and regimes to achieve adequate parasite control without requiring the development of entirely new products. Illustratively, investigations into increasing the dose of oxalic acid (Jack et al., 2021), developing long-release oxalic acid products (Maggi et al., 2016; Rodríguez Dehaibes et al., 2020), repeated acute application of oxalic acid (Berry et al., 2022) have all been trialled. Additionally, as well as combination-approaches of pairing chemical treatment with cultural management or biological opportunities apparent in beekeeping schedules have been explored (Evans et al., 2021; Gregorc et al., 2017); foe example, Al Toufailia et al. (2015, 2014) showed the potency of oxalic acid control of mites was enhanced both by minimizing brood area and by using hygienic bee breeds. Exploiting management regimes such as enforced brood-breaks during spring splits or during summer foraging dearths in combination with already established treatments remains a promising frontier in adapting current technologies to be synergistically enhanced via a cross-hierarchy use of IPM techniques (Box 1, also see Almecija et al. (2021)). Simultaneous use of multiple parasite control strategies acting to multiplicatively improve treatment success exemplifies the ‘no one silver bullet’ lesson that has emerged in varroa control reviews (Traynor et al., 2020).

### 3.2 Novel biological controls

Biological controls remain a high-demand research priority for beekeepers in treating all manner of parasites in honey bees, although the conceptualization of what counts as a biological compared to chemical control in beekeeping is a possible disconnect between practitioners and scientists; for example, beekeepers may cite organic or reduced-risk chemical controls as biological controls, counter to the typical paradigm within which IPM and wider regulatory systems typically operate. Regardless, research in these spheres remains active and in some cases already have formulated products on the market. Supplementary feeding of phytochemicals (Palmer-Young et al., 2017) mirroring the social-medication (Gherman et al., 2014; Spivak et al., 2019) that bees already undertake (e.g. resin collection (Simone-Finstrom and Spivak, 2012), or callunene consumption (Koch et al., 2019)) is a promising line of novel treatment of a variety of honey bee diseases, possibly including treatments for viruses as indicated by the success of fungal extracts shown by Stamets et al. (2018).

Microbial supplements for colony health and disease prevention are an additional novel frontier in expanding the toolkit to treat honey bee parasites (Horak et al., 2020), mirroring the exponential expansion in microbiome research across all of biological sciences. Recently identified bacterial symbionts in honey bees, such as *Lactobacillus* spp. (Tejerina et al., 2021) and *Bombella apis* (Miller et al., 2021) show promise as (micro)biological tools to help prevent or treat specific classes of parasites in beekeeping; in the case of the examples given, the suppression of fungal pathogens. Probiotic supplements for honey bee feeding are already on the market (e.g SuperDFM®-HoneyBee™ Probiotic), with initial small-scale evidence that they improve typical measures of colony vitality (Ellis, J.D. & Boncristiani, H.F. – pers. comm.) and may help in preventing bacterial outbreaks (Daisley et al., 2020; Evans and Lopez, 2004), although wider work on the exact bacteria used to treat or prevent specific disorders is likely necessary. Currently, bacteria in honey bee labelled supplements are limited to those already approved for elsewhere in agricultural use, with isolates from honey bees requiring additional legislative approval if they are to be marketed.

A subset of biological control development focusses specifically on alternatives to the need for antibiotics to treat bacterial diseases in agriculture or wider environmental settings, instead using a ‘phage-therapy’ approach (Meaden and Koskella, 2013). This extends to beekeeping, where phages targeting either EFB or AFB (foulbrood bacteria) are being explored as alternative treatment methods for these sporadically problematic bacteria. Focus has principally been on the prevention of AFB outbreaks (Brady et al., 2017; Yost et al., 2016) recently reviewed by Jończyk-Matysiak et al. (2020) and Tsourkas (2020) with newer developments from Brady et al. (2021). While these treatments show some initial promise, their adoption will depend on whether current patterns of prophylactic treatment being more effective than responsive treatment remain true, and if so, what the regulatory and economic prospects (see section 4.1 & 4.2) of application of these methods for a fairly low-problem bacterial disease will be.

Use of honey bees as model organisms for fundamental microbiological biological science (Zheng et al., 2018) has also led to the development of in-principle biological control tools. Impressive work by Leonard et al. (2020) showed that gut symbionts of honey bees could be genetically engineered to produce dsRNA of varying types and targets that assist in parasite control. For example, they demonstrate this principle by reducing viral infection and phoretic varroa lifespan (Leonard et al., 2020). Conceptually similar, although different in both development and application, is the directed evolution of possible natural enemies of parasites for deployment in living bee colonies; namely, there has been progress in the intentional evolution of varroa-infecting entomopathogenic fungi (Han et al., 2021) for deployment in bee colonies. Understandably, beekeepers receive these developments enthusiastically, although the likelihood of these lab-demonstrated mechanisms being used in-field in commercial beekeeping may be far lower than beekeepers currently understand, plausibly because of popular media coverage and opaque regulatory environments (see section 4.2).

### 3.3 Developing more chemical controls

Novel developments in bee-safe chemical control of parasite is ongoing. The major emphasis of this line of research is screening possible acaricide agents from elsewhere in agriculture or from prospective chemical analysis for use in varroa control, motivated by the challenges briefly discussed in section 2.4. These efforts span both synthetic and non-synthetic sources, including simple pharmaceuticals such as lithium salts (Kolics et al., 2021b; Stanimirovic et al., 2021; Ziegelmann et al., 2018). Essential oil research remains of interest due to low regulatory hurdles (e.g. Sabahi et al., 2017), although identification of specific active ingredients remains required for widespread uptake and ensuring bee safety; this is paralleled in the similar if smaller-scale efforts to expand treatment options for *Vairimorpha* (Burnham, 2019). For example, structural isomers of active ingredients may have different bee toxicity and are uncontrolled in their relative representations in essential oils, but can be controlled in formulated products; this is seen in comparative toxicities of thymol and carvacrol. Novel chemistry development for arthropod parasite control in beekeeping ultimately remains challenging for reasons similar to those described in section 2.1 discussing small hive beetles; in short, controlling varroa (and similar parasites) in beekeeping has been described as ‘safely killing a bug on a bug’. While a taxonomically inaccurate, if humourous, description, it pithily summarises the problem of identifying suitable differential toxicity between honey bees and varroa to trial control agents. Efforts to identify chemical treatments with suitable differential toxicity are active ongoing, with recent gains made by Bahreini et al. (2020) and Jack et al. (2021) who undertook initial differential toxicity screenings of many candidate compounds for varroa in honey bees, with some notable successes identified for forward investigation. However, despite successes in identifying possible effective agents such as lithium salts and more complex chemistries, few new products have been developed or approved in the US/EU, and even those developed may struggle in implementation.

## 4 Barriers to uptake of current and future working solutions

### 4.1 Economic barriers

**Fig. 1:**
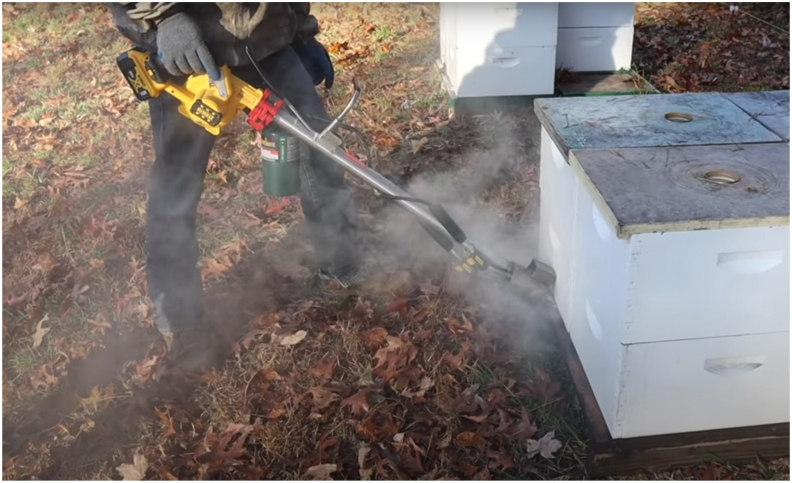
An adapted amalgamation of a leaf blower, propane gas heater, and oxalic acid vaporizer as developed by a commercial beekeeping operation to improve the economic viability of acute oxalic acid treatment of many hundreds of colonies. Devices such as this allow even large apiaries to be treated with minimal labour (relative to the wider of context of beekeeping), but rely on beekeeper-led innovation and potentially loose regulatory environments.

Any new chemistries, or control technologies in general, must be affordable on a per-colony basis for profitable beekeeping at the commercial or sideline scale. This is not a small feat, as investment in large quantities of pesticides or pharmaceuticals is a significant cost to beekeepers and may be a high-risk investment if resistance is prevalent; resistance-risks are however countered to some degree by effective screening methods, see Rinkevich (2020). In addition, while hobbyist and/or small-scale beekeepers can devote significant time and attention to each individual colony in their care, it is impractical from a labour-costs point of view to undertake certain control measures in commercial operations. This intersects with IPM (Box 1) where monitoring parasite levels in each colony quickly becomes implausible in large operations, instead requiring beekeepers to treat according to set schedules, or use indicator colonies to assess whether whole apiaries should be treated. Beekeepers remain resourceful in adapting methodology to effectively scale-up acute treatments, for example by developing efficient fast-delivery of vapourised oxalic acid (Fig. 1). Labour costs are part of the appeal of ‘place and leave’ long-release treatments such as for amitraz, thymol, and formic acid (Hansen, 2021), and limit the use of oxalic acid which has only recently seen some promise in slow-release treatments (Maggi et al., 2016). Heat-treating colonies exemplifies this barrier to uptake; there is reasonably strong evidence of the effectiveness of using temporary overheating of colonies to kill varroa (either directly, or by causing them to fall from the bee and become trapped on a sticky screen), but this approach requires each colony be heated for an extended period (Bičík et al., 2016; Kablau et al., 2020) and cannot be easily scaled up.

### 4.2 Regulatory and manufacturing barriers

While identification of possible novel treatments of bee parasites in the research environment has shown promise, the development of suitable products for regulatory approval remains a second hurdle. As discussed briefly above, developed products must be economically viable for large-scale beekeepers, meaning they are affordable but also of low labour cost to deploy (Hansen, 2021). However, the cost of registering new pesticides is high, and whilst eased somewhat in some locations for ‘reduced risk’ pesticides such as essential oils or when petition for an expansion of use of already registered control agents, there remains a need for manufacturers to fund and apply for the necessary regulatory approval for new, labelled beekeeping-deployable control agents. If a product cannot be patented, or can be easily circumvented, this technology transfer step can be a particularly challenging hurdle – especially if extensive residue analysis is required to approve treatments for use with honey supers on colonies (for example, recent changes in the USA for oxalic acid application).

One arguably pertinent example of this tech-transfer problem is the lack of progress on the development of lithium salts for varroa control, which would be an attractive, cheap, organic control method, with good contemporary evidence of its effectiveness (Kolics et al., 2021b; Stanimirovic et al., 2021). However, lithium salts are a psychoactive pharmaceutical agent used under prescription in human medicine (Lieberman and Tasman, 2006) making regulatory approval arguably even more important than for other typical agrochemicals, especially given the recent evidence of its apparent accumulation in honey bee products (Kolics et al., 2021a; Prešern et al., 2020). Further, the simple application of feed solutions including lithium salts is difficult to patent and easy for beekeepers to circumvent, reducing the prospective profitability of any labelled products where a manufacturer who funded regulatory approval needs to recoup costs. This example case demonstrates principles which apply much more widely across the sector, where laboratory and field science may be able to identify strong candidate chemicals (or other treatments as discussed above), but such findings are of little use if registered products cannot be financed, approved, and produced for beekeepers to buy at affordable rates across the hobbyist to commercial spectrum. Efforts to help alleviate this bottleneck in technology development transfer are apparent in efforts such as the IR-4 scheme in the USA (Baron et al., 2018) but remains a challenge frontier in need of better solutions.

These challenges are likely exacerbated for some biological controls (see section 3.2). Environmental releases of, for example, genetically modified insect bacteria (Leonard et al., 2020) or entomopathogenic fungi (Han et al., 2021) are likely to require significant regulatory oversight – particularly in the EU where attitudes to genetic engineering in agriculture are less tolerant. Similarly, while manufacture of these technically challenging biological solutions will be unlikely to struggle with patentability in motivating industry funding of regulatory approval, the sale costs to recover investment for manufacturers will likely be more economically prohibitive for most beekeepers then many chemical controls, which already see difficulty in legal adoption due to expense of purchase at scale (Hansen, 2021). Efforts to manufacture and then directly treat managed honey bees with RNAi fall under this category; unlike the symbiont-based approach developed by Leonard et al. (2020) discussed in section 3.2, direct RNAi has been suggested as a mechanism for control of various parasites such as *Vairimorpha* (Burnham, 2019; Paldi et al., 2010; Rodríguez-García et al., 2018), but has yet to be licensed in almost any instance for agricultural use and would face significant purchase cost barriers.

### 4.3 Cultural barriers

A subset of beekeepers will likely remain permanently opposed to certain treatment approaches, particularly the use of synthetic chemicals. This phenomenon can be linked to both operation size, but also individual philosophy as detailed by Underwood et al. (2019), and has been further characterized ‘treatment skeptic’ vs ‘treatment adherent’ by Thoms et al. (2019). The role of these treatment-skeptic beekeepers in wider bee parasite epidemiology remains an open, and possibly exciting frontier from a research perspective. On one hand, ‘unmanaged’ or ‘treatment-free’ apiaries may act as reservoirs of infection or sources of outbreaks for parasites/propagules that then threaten nearby colonies; in balance however, it is possible that having treatment-free refugia for parasites in beekeeping will slow the evolution of resistance to chemical or biological control agents; this tension is well-established when discussing the utility of refugia and chemical control resistance management (Park et al., 2015). It is possible that, with the use of adequate cultural, mechanical, and certain biological controls the latter benefit of these beekeepers may be gained without the former cost. While there are cultural barriers to the widespread and welcome uptake of some current and future control agents in honeybee parasites, the existence of a subset of beekeepers opposed to the use of specific control measures is not necessarily a problem to overall beekeeping health. Better cross-cultural and cross-industry understanding of the social science of morals and tradition in beekeeping would serve to improve our assessment of needs-gaps in parasite control in beekeeping.

## 5 Threats to bees that may need future treatments

‘Horizon scanning’ remains an active part of modern biological research, and frontiers in beekeeping parasite control do well to be on the continual look out for emerging threats, an example few of which are discussed here.

### 5.1 Invasive macroparasites

Interspecific jumps of other macroparasites in western honeybees from species native to the biodiverse *Apis* homerange of southeast Asia are being monitored closely, as the risk of a ‘new varroa’ or ‘new nosemosis’ remains of great concern to beekeepers and bee conservationists globally. A recent primer on this was penned by Ramsey (2021), who identified *Tropilaelaps* spp. and *Euvarroa* spp. as high-risk ectoparasites of *Apis* species, with the former having been observed in *Apis mellifera* in Asia for some decades (Woyke, 1984). The latter, *Euvarroa*, occupies a very similar niche to *Varroa destructor*, and so its invasive potential is open to debate – its displacement of current varroa, which occupies the same niche, would speculatively rely on an inability to vector deleterious viruses which are capable of infecting varroa (namely, DWV). However, this would significantly reduce the damage inflicted by the mite, as varroa populations without circulating vectored DWV seem to lead to low overall impact on their associated infected colonies (Brettell and Martin, 2017; Wilfert et al., 2016). Further, the acquired immunity to current acaricides in *Varroa* populations is likely lacking in *Euvarroa*, meaning an environment where abundant, heavily applied acaricides are already prevalent may make for an exceedingly difficult invasion landscape. This latter point likely applies to *Tropilaelaps* also, whereby its assumed vulnerability to currently used acaricides may inhibit its invasion potential, in a manner similar to the lack of concern over endemic tracheal mites on the basis of varroa control (Farone, 2021). Although, of note is the lifecycle of *Tropilaelaps* which spends relatively much more time on-brood than *Varroa* (de Guzman et al., 2017) potentially further reducing the effectiveness of acaricides like oxalic acid which cannot penetrate the wax cappings that protect brood. Ramsey (2021) provides an otherwise thorough introduction to these potential threats, but the need for new acaricides remains mostly unchanged given the current context of *Varroa*.

Other possible macroparasites or parasitoids have been briefly mentioned as possible new threats to honey bees, especially parasitoid phorid flies that evolved in tandem with different eusocial hymenoptera, including the ‘ant-decapitating’ flies (*Apocephalus* spp. (Core et al., 2012)) and the ‘bee-killing’ flies (*Melaloncha* spp. (Brown and Smith, 2010)). The latter were recently raised as a possible cause of major managed honey bee decline in Guatamala (G. Keller – pers. comm.). Control of parasitic/parasitoid insects in beekeeping is challenging for the same reasons outlined when discussing SHBs in section 2.1.

### 5.2 Emerging viruses

The emergence of new epidemic viruses, similar to the recent re-emergence of DWV (Wilfert et al., 2016) and arguably others (McMahon et al., 2018), remains of high risk given that viral treatments are already lacking in honey bees (see section 2.2). It is worth mention that there is significant risk of novel viral epidemic outbreaks to more industries than just beekeeping – with agriculture, livestock, conservation, and human health all arguably at the mercy of our relative inability to treat severe viral infections. Perhaps worth optimistic speculation is the observation that the currently unknown mycochemicals associated with viral suppression identified in Stamets et al. (2018) may open the possibility of bioprospecting via honey bee foraging, where discovery of novel pharmaceutical compound classes could be motivated by the observed social-medication behaviours of honey bees to treat specific infections.

### 5.3 Epidemic outbreaks of other novel microparasites

New bacteria, fungi, microsporidia, trypanosomes, and others are continually identified in honey bees, with some correlatively linked to colony decline but their possible pathological roles broadly uncharacterized, for example the recent investigations into the seemingly ubiquitous trypanosome *Lotmaria passim* (Schwarz et al., 2015). The emergence of novel directly transmitted or environmentally transmitted microparasites could devastate beekeeping in many regions; we would expect the spread of new epidemics in managed honey bees to be fast, whereby even ‘low intensity’ beekeeping is predisposed to very rapid population growth of directly transmitted or environmentally (spore-based) transmitted pathogens (Bartlett et al., 2019). In the case of bacterial outbreaks, our ability to swiftly contain infection may be determined by the prevalence of antibiotic resistance plasmids in other bacteria in honey bee colonies (Tian et al., 2012); this is an open question relatively unanswered despite the historic abuse of antibiotics in beekeeping (Box 1). For fungal outbreaks, our best speculative options may be the antifungal microbiome manipulations discussed in section 3.2, specifically *Lactobacillus* spp. (Tejerina et al., 2021) and *Bombella apis* (Miller et al., 2021). For lesser-known phyla such as microsporidia (see *Vairimorpha* section 2.0 and section 4.2) we require prospective treatment that reflects our lack of understanding of effective antimicrobials across neglected parasitic taxa in nature; however gains are being made on this frontier in beekeeping as discussed across this review spanning biotechnology approaches to plant extracts (Burnham, 2019; Rodríguez-García et al., 2021).

## 6 Concluding remarks

Efforts to improve parasite control in beekeeping will continue to gather momentum as current and future threats imperil managed honey bees. Open frontiers remain in identifying new control measures for a variety of challenging parasites that are likely to be developed by manufacturers, approved by regulators, and adopted by beekeepers. Macroparasites such as varroa still pose a serious unmet challenge to honey bee health, and microparasites such as viruses, microsporidia, and trypanosomes act as current arenas of innovative biological research. The increasingly large body of work on certain parasites and control approaches, as well the involvement of veterinary sciences, has prompted numerous strong summative reviews of aspects of parasite control in honey bees. However, more can be done to better synthesise some of the larger topics of research in this field.

## Declaration of competing interest

The sole author has no conflicts of interest to declare. No external funding was used to support this manuscript.

## Footnotes

1 ‘parasite’ will be used throughout this review as an umbrella term to refer to both pests and pathogens of all scales in honey bees/honey bee colonies, in-line with its typical use in ecology and evolutionary biology.
